# Viral caracterisation of “zoonotic” Foamy viruses

**DOI:** 10.1186/1742-4690-8-S1-A240

**Published:** 2011-06-06

**Authors:** Réjane Rua, Edouard Betsem, Sara Calattini, Antoine Gessain

**Affiliations:** 1 Epidemiology and Physiopathology of Oncogenic Retroviruses Unit, URA CNRS 3015, Pasteur Institute, Paris, 75015, France

## Background

Simian Foamy Virus is a widespread retrovirus infecting non-human primates (NHP). It is latent in PBMCs but replicates efficiently in saliva. It can be transmitted to humans mainly by bites, giving rise to a lifelong infection. Little is known about FV replication in humans. Genomic changes and quasi-species variability in human PBMCs and saliva have not been extensively studied yet.

## Materials and methods

In South Cameroon, a series of hunters bitten either by an African Green Monkey (AGM), a chimpanzee (cpz) or a gorilla (ggo) were found to be SFV-infected. Viral isolation was performed by co-cultivation of their PBMCs with BHK cells. We also analyzed quasi-species (in a 425pb-Pol fragment) from PBMCs and saliva of 9 SFVggo-infected hunters.

## Results

5 viral strains (1 SFVagm, 2 SFVcpz and 2 SFVggo) were isolated and sequenced. They are about 5-15% divergent from the corresponding prototypical sequences. Their divergence is s(ub)pecies-specific and no common genomic feature was found between the “zoonotic” strains. Quasi-species variability ranges from 0,3% in saliva to 0,5% in PBMCs. In only 2/9 cases, FV clones are clustered in two groups: PBMCs versus saliva.

## Conclusions

In contrast with previous studies, no deletion or specific mutations have been observed in the 5 “zoonotic” FV, suggesting that FV restriction in humans is not due to genetically impaired viruses. Preliminary data indicate that quasi-species variability in saliva seems not higher than in PBMCs, which might be explained by a low replication in human saliva.

